# Gender differences of the association between work-related stressors and mental health among Chinese medical professionals: a nationwide cross-sectional study

**DOI:** 10.3389/fpubh.2025.1561588

**Published:** 2025-04-08

**Authors:** Lin Chen, Lihong Wu, Jingjing Xia, Xixuan Cai, Liying Chen

**Affiliations:** Department of General Practice, Sir Run Run Shaw Hospital, School of Medicine, Zhejiang University, Zhejiang, China

**Keywords:** work-related stressor, mental health, medical professionals, gender difference, medical dispute, promotion pressure

## Abstract

**Background:**

Chinese medical professionals are under tremendous work pressure, which greatly undermines their mental health, hinders professional performance and impairs the quality of healthcare. However, the specific work-related stressor that affects mental health most and whether gender difference plays a role are not yet known. This study aims to investigate the association between work-related stressors and mental health among medical professionals in China.

**Methods:**

A cross-sectional online survey was conducted from September 29, 2022 to January 18, 2023 by recruiting 2,976 medical professionals from three representative provinces in China through purposive sampling. Anxiety and depressive symptoms were measured by the 7-item Generalized Anxiety Disorder (GAD-7) scale and Patient Health Questionnaire-9 (PHQ-9) scale. Logistic regression models were performed to identify work-related stressor significantly associated with mental health and stratified by gender.

**Results:**

The prevalence of major depressive and anxiety symptoms among Chinese medical professionals was 28.2% (839/2976) and 24.0% (714/2976), respectively. Among the subjects, 43.7% (1,302/2976) of subjects reported having ≥3 work-related stressors, which was positively related to both major depressive and anxiety symptoms. The following work-related stressors were positively associated with major depressive symptoms: violence against medical staff and promotion pressure among males; medical dispute among females. The following work-related stressors were positively associated with major anxiety symptoms: medical dispute and promotion pressure among males. While no work-related stressor showed significant association with major anxiety symptoms among females.

**Conclusion:**

These findings identified the specific work-related stressors related with the mental health, and gender differences are indicated in this relationship. Interventions directing at improving doctor-patient relationship may help to improve mental health of Chinese medical professionals. Reforming promotion system may mitigate the anxiety symptoms of male medical professionals.

## Background

Work stress is defined as an individual’s reactions to work environment characteristics that appear emotionally and physically threatening to the individual (1). Work stress has become a prevalent public health problem, exerting detrimental effects on mental health (2). Individuals facing stress with dysregulated responses may develop stress-related pathologies such as depression or anxiety disorders (3). High levels of work stress are reported to be associated with depression as well as anxiety (4, 5). Medical professionals are in risk of several mental disorders due to the exposure to accumulating work stressors including psychosocial, ergonomic and physicochemical threats (6). The negative effects of work stress on medical professions may lead to other profound influence such as hindering their professional performance and deteriorating the healthcare quality (7, 8). Therefore, growing attentions have been paid on the psychological health status of medical staff recently, warranting further research on the relationship between work stress and mental health of medical staff (9).

Several studies have assessed the mental health status of doctors in developed countries, which showed the prevalence of depressive symptoms varying from 10 to 15% (10–13). Studies focused on Chinese physicians showed different results likely due to the discrepancies of culture background and national conditions. Studies conducted in different cities in China found an estimated 18.0–25.7% of physicians with anxiety symptoms and 28.1–31.7% with depressive symptoms (14, 15). While some research indicated much higher prevalence of depressive symptoms ranging from 42.7 to 65.3% among Chinese medical staff (16–18). Some studies indicated different psychosocial stressors as risk factors for poor mental health of Chinese physicians, such as workplace violence, role insufficiency, tense doctor-patient relationship (15, 16). The outbreak of emergent public health hazards such as Corona Virus Disease-19 (COVID-19) pandemic has worsened the living condition of health care professionals even further and brought on multiple mental disorders (19). Most studies report a high prevalence of anxiety (ranging from 30 to 70%) and depressive symptoms (20–40%) among COVID-19 pandemic (20). A systemic review showed similar prevalence estimates for depression (31.8–60.5%) and anxiety (34.2–57.7%) symptoms during pandemic outbreak. The pooled prevalence of major depression disorder (MDD) was significantly higher during the pandemic (12.9–19.2%) than after (5.1–8.9%) (19).

Stress responses exhibit distinct differences across genders. The mechanisms underlying women’s heightened vulnerability to work-related mental health risks arise from an intersection of biological susceptibility, psychosocial overload, and systemic inequities (21, 22). The gender difference in stress-coping mechanisms necessitate tailored interventions to optimize workplace stress mitigation strategies. A study showed that factors like correct guidance and effective safeguards for prevention from disease transmission had larger impact on female medical staff than males during the COVID-19 outbreak (23).

Though previous studies indicated close relationship between different work-related stressors and mental health of medical professionals, most of studies investigated each stressor separately while the effect of accumulating job stressors on mental health is little explored. According to the syndemics model raised by Singer, accumulation of adverse events can create synergistic interaction: the co-occurrence of certain disease or risk factor can cause an excess burden to the population (24). A few recent studies have applied this theory to the field of work stress and mental health and found that the accumulation of job stressors increased the risk of psychological distress, indicating that job stressors may mutually enforcing the effect of each other and imposing heavy burden on employees (25–27). We are interested if the accumulation of work-related stressors may be related with the worse mental health status of Chinese medical professionals. In addition, most studies on mental health of Chinese medical staff recruited specific population as participants or conducted in one province and were in lack of gender-stratified analysis. Therefore, we carried out a nation-wide study to explore the association between work-related stressors and mental health among Chinese medical professionals and the gender difference.

## Methods

### Ethics statement

This study was approved by the Ethics Committee of Sir Run Run Shaw Hospital, affiliated with Medical College of Zhejiang University (NO. 2022–0370). All the participants read the purpose statement of the investigation and each provided an electronic informed consent. All methods were performed in accordance with the relevant guidelines and regulations.

### Participants and sampling

In this cross-sectional study, medical professionals who have direct contact with patients in medical institutions were enrolled online from September 29th, 2022 to January 18th, 2023. Inclusion criteria: ① Aged between 18 and 65 years old. ② Health technicians who have obtained the qualifications and practice certificates. ③ In-service staff in medical institutions. ⑤ Voluntarily participate in this study and sign an electronic informed consent form. Exclusion criteria: ① On vacation or sick leave for ≥2 weeks during the study. ② Hospital administrative staff.

The survey was administered via *Wenjuanxing* (a GDPR-compliant Chinese platform equivalent to Qualtrics) without collecting direct identifiers (e.g., names, ID numbers, IP addresses). Demographic variables (e.g., age, income) were collected as aggregated categories (e.g., age groups: 20–30, 31–40 years) rather than exact values to prevent re-identification. Raw datasets were permanently deleted after statistical aggregation, retaining only anonymized analytic files. Researchers signed non-disclosure agreements prohibiting unauthorized data sharing.

The sampling process was carried out as follows. Step 1: Considering the significant geographical and economic disparities across China, this study adopted purposive sampling (deliberate, non-random sampling of the target population) and selected one province from each of the eastern, central and western regions of China: economically developed Zhejiang [in eastern China with a Gross Domestic Product (GDP) of RMB 7,351.6 billion in 2021, ranked 4th nationally] (28), economically less developed Guizhou (in western China with a GDP of RMB 1,958.6 billion in 2021, ranked 22nd nationally) (29) and economically intermediate Henan (in central China with a GDP of RMB 5,888.7 billion in 2021, ranked 12th nationally) (30). Step 2: 6–8 primary medical institutions were purposively sampled in each of the three provinces, with a quota of 30–50 samples per institution; 4–6 secondary medical institutions, with a quota of 50–70 samples per institution; and 3–5 tertiary medical institutions, with a quota of 100–120 samples per institution.

The retrieval rate was 69.4% (2,996/4320). Despite the challenges posed by the COVID-19 pandemic, the response rate in this study (69.4%) aligns with international benchmarks for comparable research (31) and is consistent with response rates reported in similar studies conducted during the pandemic (32). Finally, 2,976 medical professionals were qualified for this study after excluding 20 invalid questionnaires, with an overall response rate of 68.89%.

### Measurements and instruments

The questionnaire includes four sections: socio-demographic information, lifestyles, Self-reported health status and work-related characteristics. Socio-demographic information included gender, age, education level, marital status, annual income level, and located region. Lifestyle items comprised of sedentary time, physical exercise frequency, drinking and smoking status. Work-related characteristics contained working unit level, job title, work experience, specialty, weekly working hours, night shift frequency, workload intensity and work-related stressors. Self-reported health status covered two parts: history of chronic disease and mental health. Mental health condition comprised of anxiety and depressive symptoms that are measured by the 7-item Generalized Anxiety Disorder (GAD-7) scale and the Patient Health Questionnaire-9 (PHQ-9) scale, respectively. GAD-7 scale comprises 7 items assessing the severity of anxiety symptoms over the past 2 weeks. A 4-point severity scale (0 = Not at all to 3 = Nearly every day) was employed. A total score ranging from 0 to 21 was calculated and the recommended threshold score of ≥10 was used. GAD-7 has excellent internal consistency (*α* = 0.92) and threshold score of ≥10 had a sensitivity of 89% and a specificity of 82% for generalized anxiety disorder (33). PHQ-9 scale comprises 9 items assessing the severity of depressive symptoms over the past 2 weeks. A 4-point severity scale (0 = Not at all to 3 = Nearly every day) was employed. A total score ranging from 0 to 27 was calculated and the recommended threshold score of ≥10 was used. PHQ-9 has high internal consistency (*α* = 0.84) and threshold score of ≥10 had a sensitivity of 88% and a specificity of 88% for major depression disorder (34, 35).

The work-related stressors were determined based on related articles in the literature (36, 37) and further revised by a panel of experts. To ensure content validity and contextual relevance, a focus group comprising 30 healthcare professionals was convened through stratified purposive sampling. Participants included 15 frontline physicians (from diverse departments: internal medicine, surgery, and emergency), 5 junior nurses, 5 senior nurses (ward managers with ≥5 years’ experience), 5 hospital administrators. Over three rounds of structured discussions (120 min each), their inputs informed multiple aspects:

1. Clinicians emphasized non-clinical burdens (e.g., promotion pressure, research quotas, teaching pressure) and patient distrust dynamics.
2. Nurses highlighted multitasking pressures and heavy work-load, also the direct contact to patients rises their anxiety for over expectation of patients.
3. Administrators indicated medical dispute and violence against medical staff led to increased communication costs with patients and increased anxiety among medical staff.

### Statistical analysis

All statistical analyses were performed using Statistical Package for the Social Sciences (SPSS) version 26.0 for Windows (SPSS Inc., Chicago, IL, United States). In the process of statistical analysis, continuous variables were presented as mean and standard deviation (SD). The normality of continuous variables was assessed using the Shapiro–Wilk test and visual inspection of Q-Q plots and histograms. Variables conforming to a normal distribution (*p* ≥ 0.05) were analyzed by Student’s *t*-test, while Mann–Whitney U test were applied to variables violating normality (*p* < 0.05). Categorical variables were presented as count and percentage and compared by the *χ*^2^-test.

Descriptive statistics summarized sociodemographic traits, and overall scores of PHQ-9 and GAD-7. *χ*^2^-test were used to assess the distributional differences in demographic variables among groups stratified according to number of work-related stressors. Bivariate and multivariate logistic regression models were used to evaluate the association between work-related stressors (independent variable) and anxiety/depressive symptoms (dependent variable). Demographic variables, lifestyles, and history of chronic disease were entered as covariates in multivariable logistic regression analysis and the results are presented as adjusted odds ratio (AOR). In the multivariable logistic regression analysis of specific work-related stressor, other work-related stressors were also entered as covariates. Covariates included in the models were based on *a priori* hypotheses that they would be related to anxiety and depressive symptoms. We included missing data in the analysis and marked them as ‘NA’ (not applicable) in SPSS V.26.0, with missing data accounting for less than 1%. In all statistical tests, a two-sided *p*-value <0.05 was considered significant.

## Results

There were 2,976 subjects included in the study and their demographic characteristics, work-related factors, life-related factors, depression as well as anxiety score are presented in Table 1. Of participants, 927 (31.1%) were male, 2,135 (71.7%) had master or doctor degree and 2,124(71.4%) were married. About half of participants were physicians (49.9%) and 46.6% reported to have high workload intensity. The average score of GAD-7 and PHQ-9 were 6.97 ± 4.94 and 8.00 ± 5.79, respectively.

**Table 1:** Demographic characteristics stratified by number of work-related stressors (*N* = 2,976).

Variables	Work-related stressors *N* (%)/Mean ± SD			*p*-value	Total *N* (%)/Mean ± SD
	1	2	≥3		
Gender				0.001	
Male	279 (28.2)	195 (28.6)	453 (34.8)		927 (31.1)
Female	712 (71.8)	488 (71.4)	849 (65.2)		2049 (68.9)
Age				<0.001	
<30 years	397 (40.1)	237 (34.7)	311 (23.9)		945 (31.8)
30–39 years	264 (26.6)	232 (34.0)	544 (41.8)		1,040 (34.9)
40–49 years	196 (19.8)	156 (22.8)	346 (26.6)		698 (23.5)
≥50 years	134 (13.5)	58 (8.5)	101 (7.8)		293 (9.8)
Marital status				<0.001	
Married	658 (66.4)	468 (68.5)	998 (76.7)		2,124 (71.4)
Single	306 (30.9)	200 (29.3)	275 (21.1)		781 (26.2)
Other	27 (2.7)	15 (2.2)	29 (2.2)		71 (2.4)
Education status				<0.001	
Junior and senior high school	135 (13.6)	30 (4.4)	19 (1.5)		184 (6.2)
College	297 (30.0)	138 (20.2)	222 (17.1)		657 (22.1)
Master and doctor	559 (56.4)	515 (75.4)	1,061 (81.5)		2,135 (71.7)
Annual income level (Yuan)				<0.001	
<5,000	380 (38.3)	147 (21.5)	230 (17.7)		757 (25.4)
5,000-10,000	362 (36.5)	230 (33.7)	387 (29.7)		979 (32.9)
10,001-15,000	147 (14.8)	168 (24.6)	319 (24.5)		634 (21.3)
15,001-20,000	52 (5.2)	77 (11.3)	184 (14.1)		313 (10.5)
>20,000	50 (5.0)	61 (8.9)	182 (14.0)		293 (9.8)
Job title				<0.001	
Primary	608 (61.4)	329 (48.2)	391 (30.0)		1,328 (44.6)
Intermediate	287 (29.0)	242 (35.4)	555 (42.6)		1,084 (36.4)
Senior	96 (9.7)	112 (16.4)	356 (27.3)		564 (19.0)
Working unit				<0.001	
Township health center	323 (32.6)	164 (24.0)	201 (15.4)		688 (23.1)
Community health center	166 (16.8)	110 (16.1)	110 (8.4)		386 (13.0)
County hospital	195 (19.7)	144 (21.1)	288 (22.1)		627 (21.1)
Municipal hospital	124(12.5)	113 (16.5)	333 (25.6)		570 (19.2)
Provincial hospital	104 (10.5)	109 (16.0)	321 (24.7)		534 (17.9)
Others	79 (8.0)	43 (6.3)	49 (3.8)		171 (5.7)
Work experience (years)	11.55 ± 10.60	10.92 ± 9.00	12.00 ± 8.61	<0.001	11.60 ± 9.41
Specialty				<0.001	
Physician	380 (38.3)	323 (47.3)	783 (60.1)		1,486 (49.9)
Nurse	288 (29.1)	205 (30.0)	313 (24.0)		806 (27.1)
Others	323 (32.6)	155 (22.7)	206 (15.8)		684 (23.0)
Weekly working hours				<0.001	
<40	298 (30.1)	136 (19.9)	143 (11.0)		577 (19.4)
40–50	327 (33.0)	281 (41.1)	490 (37.6)		1,098 (36.9)
51–60	124 (12.5)	114 (16.7)	292 (22.4)		530 (17.8)
>60	242 (24.4)	152 (22.3)	377 (29.0)		771 (25.9)
Workload intensity				<0.001	
Low	49 (4.9)	17 (2.5)	19 (1.5)		85 (2.9)
Medium	597 (60.2)	383 (56.1)	525 (40.3)		1,505 (50.6)
High	345 (34.8)	283 (41.1)	758 (58.2)		1,386 (46.6)
Night shift					
Never	275 (27.7)	137 (20.1)	185 (14.2)		597 (20.1)
1–6 times per half-year	125(12.6)	83 (12.2)	147 (11.3)		355 (11.9)
2–4 times per month	205 (20.7)	171 (25.0)	374 (28.7)		750 (25.2)
2–3 times per week	267 (26.9)	221 (32.4)	504 (38.7)		992 (33.3)
Over 3 times per week	119 (12.0)	71 (10.4)	92 (7.1)		282 (9.5)
Exercise frequency				<0.001	
Everyday	82 (8.3)	26 (3.8)	37 (2.8)		145 (4.9)
5–7 times per week	59 (6.0)	23 (3.4)	34 (2.6)		116 (3.9)
1–4 times per week	218 (22.0)	176 (25.8)	269 (20.7)		663 (22.3)
1–3 times per month	340 (34.3)	243 (35.6)	536 (41.2)		1,119 (37.6)
Never	292 (29.5)	215 (31.5)	426 (32.7)		933 (31.4)
Hours of sedentary time				0.002	
≤2	166 (16.8)	115 (16.8)	175 (13.4)		456 (15.3)
2–4	199 (20.1)	143 (20.9)	218 (16.7)		560 (18.8)
4–6	259 (26.1)	171 (25.0)	334 (25.7)		764 (25.7)
6–8	220 (22.2)	131 (19.2)	332 (25.5)		683 (23.0)
≥8	147 (14.8)	123 (18.0)	243 (18.7)		513 (17.2)
Cigarette smoking				0.031	
Yes	124 (12.5)	58 (8.5)	137 (10.5)		319 (10.7)
No	867 (87.5)	625 (91.5)	1,165 (89.5)		2,657 (89.3)
Alcohol drinking				<0.001	
Yes	266 (26.8)	152 (22.3)	401 (30.8)		819 (27.5)
No	725 (73.2)	531 (77.7)	901 (69.2)		2,157 (72.5)
Chronic disease				<0.001	
0	581 (58.6)	387 (56.7)	605 (46.5)		1,573 (52.9)
1	215 (21.7)	138 (20.2)	265 (20.4)		618 (20.8)
2	107 (10.8)	85 (12.4)	191 (14.6)		383 (12.9)
≥3	88 (8.9)	73 (10.7)	241 (18.5)		402 (13.5)
PHQ-9 score	6.67 ± 5.57	7.39 ± 5.32	9.34 ± 5.91	<0.001	8.00 ± 5.79
GAD-7 score	5.79 ± 4.77	6.32 ± 4.49	8.22 ± 5.00	<0.001	6.97 ± 4.94

The associations between demographic characteristics and numbers of work-related stressors were assessed (Table 1). The results revealed significant association of all the factors included in the study and numbers of work-related stressors (*p*<0.05).

Table 2 shows the results of the bivariate analysis for the association of job-related factors and other covariates with mental health status for medical professionals. The following factors were found to be associated with a higher risk of depressive and anxiety symptoms for the participants (*p*<0.05): intermediate job title, working in provincial hospital, having multiple work-related stressors, prolonged working hours, high workload intensity, frequent night shift, lack of exercise, smoking, chronic disease history. Drinking was associated with depression only (*p* = 0.037) and age was associated with anxiety (*p* < 0.001).

**Table 2:** Bivariate analysis of the relationships between occupational factors and covariates and mental health among medical professionals (*N* = 2,976).

Variables	Depression				Anxiety			
	Yes *N* (%)	No *N* (%)	OR (95%CI)	*p*-value	Yes *N* (%)	No *N* (%)	OR (95%CI)	*p*-value
Gender								
Male	270 (32.6)	657 (30.6)	1.00		241 (33.7)	686 (30.3)	1.00	
Female	559 (67.4)	1,490 (69.4)	0.913 (0.769–1.084)	0.299	474 (66.3)	1,575 (69.7)	0.857 (0.716–1.025)	0.090
Age								
<30 years	253 (30.5)	692 (32.2)	1.00		198 (27.7)	747 (33.0)	1.00	
30–39 years	319 (38.5)	721 (33.6)	1.210 (0.996–1.471)	0.055	300 (42.0)	740 (32.7)	**1.529 (1.244–1.880)**	**<0.001**
40–49 years	192 (23.2)	506 (23.6)	1.038 (0.833–1.293)	0.740	160 (22.4)	538 (23.8)	1.122 (0.886–1.421)	0.339
≥50 years	65 (7.8)	228 (10.6)	0.780 (0.571–1.064)	0.117	57 (8.0)	236 (10.4)	0.911 (0.656–1.266)	0.580
Job title								
Primary	329 (39.7)	999 (46.5)	1.00		289 (40.4)	1,039 (46.0)	1.00	
Intermediate	341 (41.1)	743 (34.6)	**1.394 (1.165–1.666)**	**<0.001**	289 (40.4)	1,039 (35.2)	**1.307 (1.084–1.576)**	**0.005**
Senior	159 (19.2)	405 (18.9)	1.192 (0.955–1.488)	0.120	137 (19.2)	427 (18.9)	1.153 (0.914–1.455)	0.229
Working unit								
Township health center	177 (21.4)	511 (23.8)	1.00		151 (21.1)	537 (23.8)	1.00	
Community health center	90 (10.9)	296 (13.8)	0.878 (0.656–1.175)	0.381	73 (10.2)	313 (13.8)	0.829 (0.607–1.133)	0.240
County hospital	181 (21.8)	446 (20.8)	1.172(0.919–1.494)	0.201	153 (21.4)	474 (21.0)	1.148 (0.888–1.484)	0.292
Municipal hospital	153 (18.5)	417 (19.4)	1.059 (0.823–1.363)	0.654	139 (19.4)	431 (19.1)	1.147 (0.882–1.492)	0.307
Provincial hospital	185 (22.3)	349 (16.3)	**1.530 (1.195–1.959)**	**0.001**	155 (21.7)	379 (16.8)	**1.454 (1.122–1.886)**	**0.005**
Others	43 (5.2)	128 (6.0)	0.970 (0.660–1.426)	0.876	44 (6.2)	127 (5.6)	1.232 (0.836–1.815)	0.291
Work-related stressors								
1	194 (23.4)	797 (37.1)	1.00		172 (24.1)	819 (36.2)	1.00	
2	170 (20.5)	513 (23.9)	**1.361 (1.077–1.720)**	**0.010**	122 (17.1)	561 (24.8)	1.036 (0.802–1.337)	0.789
≥3	465 (56.1)	837 (39.0)	**2.282 (1.881–2.770)**	**<0.001**	421 (58.9)	881 (39.0)	**2.275 (1.861–2.783)**	**<0.001**
Weekly working hours								
<40	117 (14.1)	460 (21.4)	1.00		90 (12.6)	487 (21.5)	1.00	
40–50	262 (31.6)	836 (38.9)	1.232 (0.964–1.576)	0.096	205 (28.7)	893 (39.5)	1.242 (0.947–1.629)	0.117
51–60	166 (20.0)	364 (17.0)	**1.793 (1.364–2.357)**	**<0.001**	159 (22.2)	371 (16.4)	**2.319 (1.732–3.104)**	**<0.001**
>60	284 (34.2)	487 (22.7)	**2.293 (1.785–2.945)**	**<0.001**	261 (36.5)	510 (22.6)	**2.769 (2.114–3.627)**	**<0.001**
Workload intensity								
Low	14 (1.7)	71 (3.3)	1.00		13 (1.8)	72 (3.2)	1.00	
Medium	310 (37.4)	1,195 (55.7)	1.316 (0.732–2.365)	0.359	240 (33.6)	1,265 (55.9)	1.051 (0.573–1.927)	0.873
High	505 (60.9)	881 (41.0)	**2.907 (1.622–5.210)**	**<0.001**	462 (64.6)	924 (40.9)	**2.769 (1.518–5.051)**	**0.001**
Night shift								
Never	138 (16.6)	459 (21.4)	1.00		114 (15.9)	483 (21.4)	1.00	
1–6 times per half-year	96 (11.6)	259 (12.1)	1.233 (0.912–1.667)	0.174	87 (12.2)	268 (11.9)	**1.375 (1.002–1.887)**	**0.048**
2–4 times per month	172 (20.7)	578 (26.9)	0.990 (0.767–1.278)	0.937	151 (21.1)	599 (26.5)	1.068 (0.814–1.401)	0.634
2–3 times per week	320 (38.6)	672 (31.3)	**1.584 (1.256–1.998)**	**<0.001**	275 (38.5)	717 (31.7)	**1.625 (1.269–2.080)**	**<0.001**
Over 3 times per week	103 (12.4)	179 (8.3)	**1.914 (1.406–2.605)**	**<0.001**	88 (12.3)	194 (8.6)	**1.922 (1.390–2.658)**	**<0.001**
Exercise frequency								
Everyday	19 (2.3)	126 (5.9)	1.00		21 (2.9)	124 (5.5)	1.00	
5–7 times per week	19 (2.3)	97 (4.5)	1.299 (0.652–2.587)	0.457	11 (1.5)	105 (4.6)	0.619 (0.285–1.342)	0.224
1–4 times per week	128 (15.4)	535 (24.9)	1.587 (0.944–2.667)	0.082	110 (15.4)	553 (24.5)	1.175 (0.708–1.948)	0.533
1–3 times per month	306 (36.9)	813 (37.9)	**2.496 (1.514–4.115)**	**<0.001**	271 (37.9)	848 (37.5)	**1.887 (1.165–3.057)**	**0.010**
Never	357 (43.1)	576 (26.8)	**4.110 (2.493–6.777)**	**<0.001**	302 (42.2)	631 (27.9)	**2.826 (1.745–4.578)**	**<0.001**
Cigarette smoking								
Yes	113 (13.6)	206 (9.6)	**1.487 (1.164–1.900)**	**0.001**	105 (14.7)	214 (9.5)	**1.647 (1.282–2.115)**	**<0.001**
No	716 (86.4)	1941 (90.4)	1.00		610 (85.3)	2047 (90.5)	1.00	
Alcohol drinking								
Yes	251 (30.3)	568 (26.5)	**1.207 (1.012–1.440)**	**0.037**	216 (30.2)	603 (26.7)	1.190 (0.989–1.432)	0.065
No	578 (69.7)	1,579 (73.5)	1.00		499 (69.8)	1,658 (73.3)	1.00	
Chronic disease								
0	339 (40.9)	1,234 (57.5)	1.00		316 (44.2)	1,257 (55.6)	1.00	
1	170 (20.5)	448 (20.9)	**1.381 (1.116–1.710)**	**0.003**	140 (21.1)	478 (19.6)	1.165 (0.930–1.459)	0.184
2	138 (36.0)	245 (64.0)	**2.050 (1.612–2.608)**	**<0.001**	117 (16.4)	266 (11.8)	**1.750 (1.363–2.247)**	**<0.001**
≥3	182 (22.0)	220 (10.2)	**3.011 (2.392–3.791)**	**<0.001**	142 (19.9)	260 (11.5)	**2.173 (1.711–2.759)**	**<0.001**

Depression cases are defined by the threshold score of ≥ 10. Anxiety cases are defined by the threshold score of ≥ 10. *p* values and AOR in bold are statistically significant.

Table 3 shows the results of multivariate analysis evaluating the association of work-related factors with mental health status adjusted for other covariates. Having ≥3 work-related stressors turned to be the only work-related factor significantly related to a higher risk for both depression (AOR 1.815, 95% CI = 1.447–2.278, *p* < 0.001) and anxiety symptoms (AOR 1.862, 95% CI = 1.469–2.359, *p* < 0.001). Having 2 work-related stressors (AOR 1.297, 95% CI = 1.007–1.670, *p* = 0.044), high workload intensity (AOR 1.934, 95% CI = 1.013–3.694, *p* = 0.046) were significantly associated with an increased risk of having depression symptoms. Working 51–60 h (AOR 1.418, 95% CI = 1.025–1.963, *p* = 0.035) or > 60 h (AOR 1.523, 95% CI = 1.110–2.091, *p* = 0.009) per week was significantly associated with an increased risk of having anxiety symptoms.

**Table 3:** Multivariate logistic regression for work-related factors associated with mental health among medical professionals (*N* = 2,976).

Work-related factors	Depression		Anxiety	
	AOR (95%CI)	*p*-value	AOR (95%CI)	*p*-value
Work-related stressors				
1	1.00		1.00	
2	**1.297 (1.007–1.670)**	**0.044**	1.019 (0.774–1.341)	0.893
≥3	**1.815 (1.447–2.278)**	**<0.001**	**1.862 (1.469–2.359)**	**<0.001**
Weekly working hours				
<40	1.00		1.00	
40–50	0.950 (0.728–1.239)	0.704	0.949 (0.710–1.269)	0.726
51–60	1.064 (0.782–1.449)	0.691	**1.418 (1.025–1.963)**	**0.035**
>60	1.295 (0.961–1.745)	0.089	**1.523 (1.110–2.091)**	**0.009**
Workload intensity				
Low	1.00		1.00	
Medium	1.139 (0.603–2.152)	0.688	0.954 (0.492–1.849)	0.888
High	**1.934 (1.013–3.694)**	**0.046**	1.905 (0.973–3.371)	0.060
Night shift				
Never	1.00		1.00	
1–6 times per half-year	1.008 (0.728–1.395)	0.963	1.160 (0.825–1.632)	0.393
2–4 times per month	**0.702 (0.527–0.935)**	**0.015**	0.741 (0.547–1.005)	0.053
2–3 times per week	0.957 (0.731–1.251)	0.746	0.910 (0.685–1.210)	0.517
Over 3 times per week	1.392 (0.980–1.977)	0.065	1.192 (0.825–1.724)	0.350

Depression cases are defined by the threshold score of ≥ 10. Anxiety cases are defined by the threshold score of ≥ 10. *p* values and AOR in bold are statistically significant.

The results in Table 3 indicated the importance of having multiple work-related stressors on mental health of medical professionals. Further analyses were made to evaluate the association of each work-related stressor with mental health status for all the participants (Table 4) as well as each gender, respectively (Table 5). Medical dispute and promotion of professional title were found to be significantly associated with an increased risk of both depression and anxiety symptoms. Violence against medical staff and promotion of professional titles were found to be significantly associated with an increased risk of depression symptoms among male medical professionals. Medical dispute and promotion of professional titles were found to be significantly associated with an increased risk of anxiety symptoms among male medical professionals. Medical dispute was found to be significantly associated with an increased risk of depression symptoms among female medical professionals.

**Table 4:** Multivariate logistic regression for each work-related stressor associated with mental health among medical professionals (*N* = 2,976).

Work-related stressors	Depression	Anxiety		
	AOR (95%CI)	*p*-value	AOR (95%CI)	*p*-value
Heavy clinical workload	0.983 (0.763–1.267)	0.897	1.072 (0.820–1.402)	0.611
Medical dispute	**1.301 (1.024–1.653)**	**0.031**	**1.333 (1.039–1.709)**	**0.024**
Violence against medical staff	1.230 (0.905–1.670)	0.185	1.195 (0.871–1.639)	0.270
Over expectation of patients	0.895 (0.714–1.121)	0.335	0.943 (0.745–1.194)	0.627
Promotion pressure	**1.317 (1.041–1.668)**	**0.022**	**1.337 (1.044–1.711)**	**0.021**
Scientific research pressure	0.849 (0.714–1.121)	0.217	1.028 (0.785–1.345)	0.843
Teaching pressure	1.084 (0.844–1.393)	0.526	1.040 (0.801–1.350)	0.770
Others	1.131 (0.896–1.429)	0.300	**1.330 (1.044–1.694)**	**0.021**

Depression cases are defined by the threshold score of ≥ 10. Anxiety cases are defined by the threshold score of ≥ 10. *p* values and AOR in bold are statistically significant.

**Table 5:** Multivariate logistic regression for each work-related stressor and gender differences in relation to mental health (*N* = 2,976).

Work-related stressors	Male				Female			
	Depression		Anxiety		Depression		Anxiety	
	AOR (95%CI)	*p*-value	AOR (95%CI)	*p*-value	AOR (95%CI)	*p*-value	AOR (95%CI)	*p*-value
Heavy clinical workload	1.114 (0.686–1.810)	0.662	1.318 (0.803–2.162)	0.275	0.925 (0.683–1.253)	0.613	0.971 (0.701–1.345)	0.859
Medical dispute	1.110 (0.724–1.703)	0.632	**1.677 (1.090–2.581)**	**0.019**	**1.367 (1.016–1.838)**	**0.039**	1.171 (0.855–1.602)	0.325
Violence against medical staff	**2.038 (1.175–3.535)**	**0.011**	1.477 (0.853–2.557)	0.164	0.970 (0.666–1.412)	0.872	1.066 (0.719–1.581)	0.751
Over expectation of patients	0.866 (0.574–1.306)	0.492	0.973 (0.641–1.478)	0.898	0.941 (0.714–1.240)	0.665	0.981 (0.733–1.312)	0.895
Promotion pressure	**1.581 (1.026–2.436)**	**0.038**	**1.837 (1.179–2.863)**	**0.007**	1.250 (0.937–1.668)	0.130	1.142 (0.841–1.550)	0.394
Scientific research pressure	0.764 (0.475–1.227)	0.265	0.764 (0.473–1.235)	0.272	0.848 (0.617–1.166)	0.310	1.139 (0.815–1.592)	0.446
Teaching pressure	1.097 (0.709–1.699)	0.677	1.160 (0.742–1.814)	0.516	1.061 (0.777–1.449)	0.710	0.980 (0.706–1.361)	0.905
Others	1.111 (0.707–1.747)	0.648	1.532 (0.976–2.406)	0.064	1.154 (0.874–1.524)	0.313	1.249 (0.932–1.674)	0.137

Depression cases are defined by the threshold score of ≥ 10. Anxiety cases are defined by the threshold score of ≥ 10. *p* values and AOR in bold are statistically significant.

## Discussion

### Prevalence of anxiety and depressive symptoms among Chinese medical professionals

Our study has shown that anxiety and depression symptoms were common among Chinese medical professionals based on a nationwide cross-sectional study. In addition, our study explored the association between different work-related stressors and mental health of Chinese medical professionals.

The prevalence of major depressive symptoms (28.2%) and anxiety symptoms (24.0%) observed in our study substantially exceeds rates reported in both Chinese and global general populations. Prior to the COVID-19 pandemic, population-based studies using PHQ-9 thresholds equivalent to our criteria documented depression rates of 7.0% in China (38) and 12.5% worldwide (39). Notably, even during the COVID-19 pandemic—a period associated with heightened psychological distress—a nationally representative Chinese study reported depression and anxiety rates of only 10.8 and 10.0%, respectively, in the general population (40), emphasizing the disproportionate mental health burden of healthcare workers. The COVID-19 pandemic has exacerbated this vulnerability: systematic reviews indicate that healthcare professionals showed high prevalence of anxiety (31.9%) and depressive symptoms (31.4%) (41). Studies using same assessment scales and threshold showed 5.09–32.76% anxiety symptoms among health care professionals during pandemic (42). Previous studies further indicated a potential prolonged mental health deterioration of healthcare workers even after acute outbreak phases in COVID-19 as well as previous pandemics (e.g., SARS, MERS) (19, 43).

The prevalence rate of major depressive symptom in our study (28.2%) was higher than that reported in a Liaoning-based study among medical residents (12.90%) with the same evaluation scale (18). Our study also showed a much higher prevalence of major anxiety symptoms (24.0%) than that in another study (9.1%) done by the same research team (44). However, another study also conducted in Liaoning province indicated a much higher prevalence of depressive symptoms among Chinese doctors (65.3%) by using the Chinese version of the Center for Epidemiologic Studies Depression Scale (CES-D) to measure depressive symptoms (16). The divergent results among studies may due to the differences of demographic data such as specialty, job title, and place of residence, as well as the study sampling method and the assessment scale. Pandemic timing also likely contributes to these differences. Our result underscore the need for longitudinal monitoring given evidence that psychological sequelae often persist post-crisis and the urgency of institutional mental health supports tailored to medical professionals, particularly in pandemic recovery phases.

### Work-related factors related to mental health among Chinese medical professionals

Several work-related factors were included in our study and turned out to be significantly associated with mental health of medical professionals. The results showed that medical workers who work 61–70 h per week were at greater risk of anxiety and depressive symptoms. This result is in accordance with previous studies indicating a positive correlation between lengthy working hours and worse mental health condition of physicians among different countries (15, 45, 46). This study also showed that higher work intensity was a significant risk factor for major depressive symptoms (AOR 1.934, 95%CI 1.013–3.694), which is in consistent with the findings of previous studies (47, 48). These results indicated that hospital managers should formulate proper strategies to control the working hours within a reasonable range and reduce the work intensity to some extent to protect the mental health of medical staff.

Besides common work-related factors, medical professionals experience some specific work stressors as a special occupational group adding extra working pressure (6). The results showed that the accumulation of ≥3 work-related stressors turned to be the only work-related factor significantly increased the risk of both major depression symptoms and anxiety symptoms. This result shown in Table 3 indicated that psychosocial work stressor exerts more influence on mental health than other work-related factors (including weekly working hours, workload intensity and night shift frequency). For depression symptoms, the incremental exposure to work-related stressor is linked to worse mental conditions. Syndemics model raised by Singer indicates adverse interaction among multiple social conditions, contributing to the formation, clustering, and spread of disease (24). Multiple work-related stressors included in the study may exert syndemic effects on healthcare workers’ mental health through the following synergistic mechanisms:1. Excessive workloads may compromise clinicians’ capacity to manage patient-doctor conflicts effectively, thereby triggering a vicious cycle of strained relationships and violence against medical staff. 2. China’s healthcare system architecture mandates simultaneous fulfillment of clinical, academic, and research responsibilities. Due to the competing demands of these professional domains, it is hard for most medical professionals to keep a good balance. 3. Syndemic interactions exhibit temporally compounding characteristics. For instance, the convergence of acute stressors (e.g., violence against medical staff) with chronic occupational burnout from heavy clinical work load culminates in worsened mental health status. Previous study also showed that the accumulation of several work stressors is associated with low work ability (27) and higher risk of disability pensions (49), exerting more detrimental effects than single stressor. This study supported the findings of previous studies and underlined the need for more research on this topic.

### The correlation between specific work-related stressor and mental health and the gender differences involved

This study explored the relationship between specific work-related stressor and mental health status among Chinese medical professionals. Results showed that medical dispute and promotion of professional title were significantly associated with a higher risk of major anxiety symptoms and major depression symptoms among Chinese medical professionals. The huge population basis and increasing health needs led to the heavy load for Chinese doctors and growing medical disputes, which may worsen the doctor-patient relationship. The intense doctor-patient relationship increased the chance of having depressive and anxiety symptoms based on previous study (15, 16), which is in consistent with the conclusions of this study. This alarming status resulted in violence against medical workers and attracted widespread attention (50), calling for multiple improvement measures including but not limited to reform of the medical system, policy intervention, and the strengthening of doctor-patient communication skills.

Promotion of professional titles turned out to be another source of burden significantly associated with the mental health of physicians, which is less mentioned in previous research. The professional title promotion system in China is consisted of several different indexes including clinical workload, teaching tasks as well as scientific research output. The specific requirements for each index vary among different working units and scientific requirement is especially demanding for physicians who work in tertiary hospitals. Researchers interviewed health care providers from HIV/AIDS clinics in Guangxi and found that research grants and publications were two most difficult criteria for promotion (36). The exorbitant scientific demand was regarded as an important driving force upon scientific misconduct by 65.90% researchers in tertiary hospitals in China (51). Further studies are warranted to evaluate the difficulties that doctors face in the promotion process and provide intervention measures.

Differences in the associations between specific work-related stressor and metal health were found between genders, confirming the importance of performing gender-stratified analysis. Medical dispute turned out to be relevant with the anxiety symptoms among males and depressive symptoms among females, indicating its impact on mental health of Chinese medical professionals for both genders though in different aspect. The difference of impact on mental health may be due to the divergence of psychological defense mechanisms between genders (52), emanating from biomedical, psychosocial as well as epidemiological perspective (53, 54). Physiologically, fluctuations in sex hormones and associated neurotransmitter dynamics may predispose females to prolonged rumination when confronting medical dispute and increase susceptibility to depression. While males exhibit sustained hypothalamic–pituitary–adrenal (HPA) axis activation under chronic stress, leading to prolonged cortisol elevation. When exposed to uncontrollable occupational stressors like medical dispute, this physiological response may enhance maladaptive anxiety-related behavioral pattern. Psychosocially, female medical professionals are often expected to show more empathy, accelerating emotional exhaustion when facing medical dispute. Moreover, the internalization of medical dispute as personal failure aligns closely with the cognitive triad of depression (negative self-view, hopelessness, and catastrophizing). While males may initially externalize stressors through attributions to systemic flaws, challenges to their socially constructed role as “authoritative care providers” can trigger identity-threatening anxiety. Previous studies also showed that workplace violence was a significant risk factor for depression among females rather than males (55). Female health-care workers showed higher prevalence of both depressive and anxiety symptoms than males during COVID-19 pandemic (56). The results of previous studies and this paper suggest that more in-depth research on gender disparities and targeted interventions for the mental health of healthcare workers are needed.

While promotion pressure was indicated as risk factor for both depressive and anxiety symptoms among males only. Traditional Chinese culture assumes men to be chief breadwinners, working outside the household to earn income for the family (57). A Confucian principle manifests this sexual division of labor: “Men are primarily outside the home, women are primarily inside the home” (58). Though China’s female labor force participation rate is higher than the world average level, this traditional division of labor makes it more of a burden for men to bear more economic pressures. The economic pressure then turns into promotion pressure since the income level is directly linked to job title.

Therefore, male and female medical professionals might both perceive work stressors differently and also develop different coping strategies due to the differing gender social role expectations, indicating that hospital administrators should develop appropriate measures for males and females, respectively, to improve the mental health of medical staff.

### Limitations of the study and future research prospects

Several limitations of the present study should be taken into consideration. First of all, as a cross-sectional study, the causality between anxiety or depressive symptoms and their related risk factors cannot be established based on this study. Future longitudinal studies are warranted to verify the conclusions drawn from our study. Furthermore, several influential factors of mental health failed to be measured and adjusted in this study including number of children, personality traits, earlier psychiatric morbidity, family history and life events. In addition, this study focused on exploring the impact of work-related psychosocial factors on mental health. It would be more comprehensive if work-related social support from coworkers and work-related social support from family could be incorporated in further studies. Besides, there are some mediating variables between work stress and mental health like burnout (18, 44), resilience, psychological capital (59) reported in previous studies. These potential mediating factors were not measured in this study, which requires future research and exploration. The selection biases of voluntary online surveys should also be taken into consideration. The work-related stressors we focused in this study are self-reported, reflecting subjective perceptions of the work environment as well as the participants’ willingness to highlight them. Findings in this study also might not fully apply to other countries due to differences in social vibes, anthropology, and cultural backgrounds. Finally, the anxiety and depression condition are measured by self-reporting scale instead of clinical diagnosis made by psychiatrists. However, the scales we used in this study has been verified and widely applied in previous studies, which increases the validity of the conclusion.

## Conclusion

Our study showed that about a quarter of Chinese medical professionals had depressive and anxiety symptoms. Having at least 3 work-related stressors was significantly associated with worse mental status. There are gender differences in the specific work-related stressor that affect the mental status. Medical dispute and promotion of professional title were shown to significantly increase the risk of getting anxiety and depressive symptoms. Therefore, strategies aiming at improving doctor-patient relationship and polices contribute to the establishment of a reasonable promotion system should be paid more attention, which will strongly improve the mental health of Chinese medical professionals.

## Data Availability

The original contributions presented in the study are included in the article/supplementary material, further inquiries can be directed to the corresponding author.
